# Supplementary material for: Protruding organic surfaces triggered by in-plane electric fields
Source: Nat Commun. 2017 Nov 15;8:1526. doi: 10.1038/s41467-017-01448-w (PMC5688145; doi:10.1038/s41467-017-01448-w)
Supplement: Supplementary file 1 — Supplementary Information [file 41467_2017_1448_MOESM1_ESM.pdf]

Supplementary Note 1: Principle of DHM

Time resolved surface topography is measured using a Digital Holography Microscopy from Lyncée Tec SA (www.lynceetec.com). The principle of the DHM to form a hologram is to allow two coherent beams (capable of forming interference) with slightly different propagation directions to interfere. This principle is illustrated in Supplementary Figure1. The collimated (paralleled) source beam (laser diode), is separated into two: the object beam O and the reference beam R. The object beam illuminates the sample via the objective. The retro diffused beam is collected by the objective of the microscope, then recombined with the reference beam to form a hologram in the camera. [1] A computer program convert the holographic data to a 3D image of the surface.

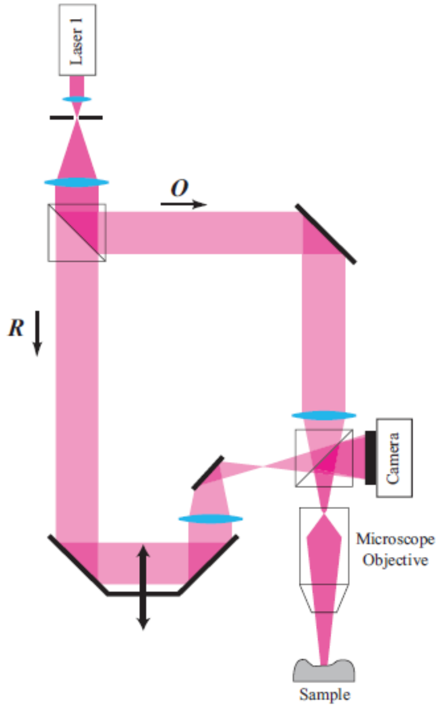

Supplementary Figure 1. Simplified optical principle of a DHM in reflection: the beam source, laser diode, is separated into two: an object beam O, and a reference beam R. The object beam illuminates the sample via the condenser. The retro diffused wave front is collected by the objective, then recombined with the reference beam to form a hologram in the camera. The

length of the reference beam must be adjusted to be the same as that of the object beam by moving the mirror unit M. Figure is provided by Lyncée Tec SA.

Supplementary Note 2: Surface deformations upon electric actuation

Figure 2A in the main text is just one example of a measurement. To check on reproducibility, many samples were made which all show similar, but sometimes slightly deviating because of the initial state of the coating. Supplementary Figure 2 show some typical other measurements, all with dynamic protrusions of the order of 100 nm.

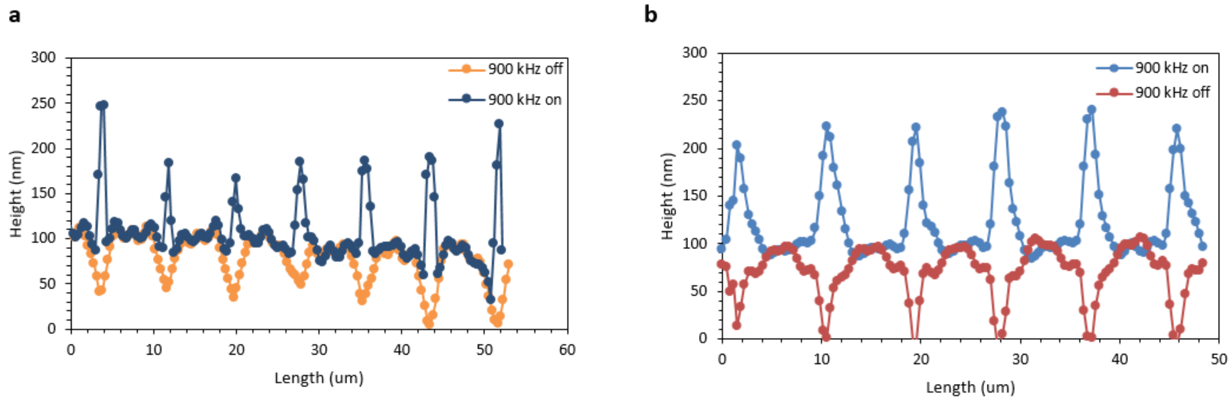

Supplementary Figure 2. Cross-section of the surface profiles measured at a random selection of samples made in accordance to the procedure described in the Methods section.

Supplementary Note 3: Dielectric measurements

To estimate whether the electrical energy is coupled to the LCNs efficiently, we modelled the distribution of the electric field between the electrodes. It is found that due to the high dielectric permittivity (>17, Supplementary Figure 3a) of the LCN, the field is mainly passing through the LCNs, with little loss of energy to the underlying substrate or the air above.

The dielectric properties are quantified. For example, the energy from the external electric field stored in the material is characterized by the real part of permittivity (ε’). The loss factor (ε’’) measures energy dissipation or loss as a material placed in an external electric field. Broadband Dielectric Spectroscopy (BDS) is employed to study the relaxation dynamics of the LCN. BDS measurements were performed isothermally in the frequency range 10^{-2} Hz to 10^{6} Hz, using a high-resolution dielectric analyzer (Alpha Analyzer, Novocontrol Technologies) in the temperature range from 25 to 100 °C. Supplementary Figure 3b shows the frequency dependent permittivity and dielectric loss of the LCNs film under an applied AC electric field at various temperatures.

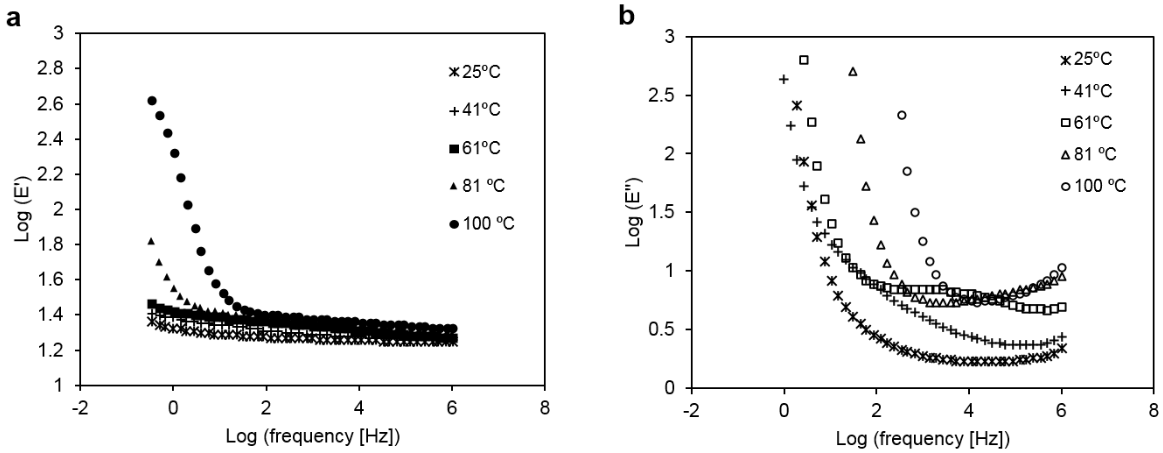

Supplementary Figure 3. Temperature dependent dielectric properties of the LCNs. (a) Permittivity measured from 0.1 Hz to 1 MHz. (b) Time dependent dielectric loss.

Supplementary Note 4: Sample heating and thermal effects

The input voltage and the corresponding output current measured from the circuit (Fig. 1f) are shown in Supplementary Figure 4a. When applying an AC voltage of 75 V the current in the

circuit is measured as 500 μA. The power consumed in the circuit is calculated as: $P_{avg} = V_{max} I_{max} \cos\varphi$, where φ is the phase lag between voltage and current which is derived from Supplementary Figure 4a to be 5.5. With this information we estimate the approximate temperature rise under adiabatic conditions from $Q = P \times t = C_p \times m \times \Delta T$, where Q is the energy that heats the sample, t is the length of time that the electric field is on, C_p is the specific heat, m is the mass of the sample, and ΔT is the temperature increase. C_p is estimated as 1550 (J/ kg·K) (http://www.engineeringtoolbox.com/specific-heat-polymers-d_1862.html). If the electric field is turned on for 30s, the calculated temperature increase is 8 ^{o}C.

The temperature change during electric actuation is also monitored in real-time by an infrared camera. From Supplementary Figure 4b we can see that the temperature increases 6 ^{o}C, from 26 ^{o}C to 32 ^{o}C, within 30 seconds, very near our calculated estimate. After 30 seconds the temperature stabilises because of heat emission due to conduction and radiation.

The relatively low temperature increase suggests minor deformation of the surface of the coating due to heat generated in the circuit; Based linear thermal expansion data known for LCNs[2] we estimate this to be 6.4 nm.

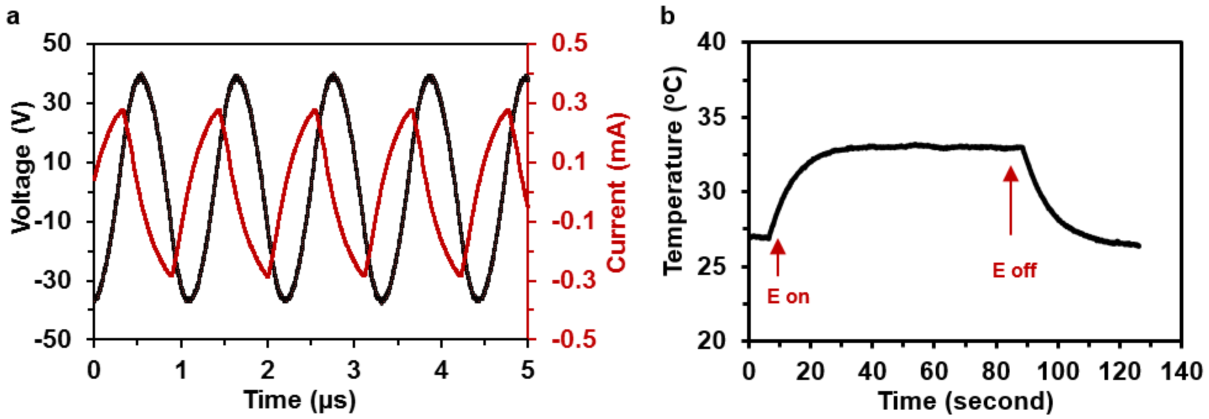

Supplementary Figure 4. Temperature rise on the sample induced by the circuit. a, The V-I phase diagram of the circuit. b, Measured temperature induced by the electric field (75 V, 900 kHz).

Supplementary Note 5: Dynamic Mechanical Thermal Analysis (DMTA)

The mechanical properties of the free standing liquid crystal polymer films with homeotropic alignment were measured by Dynamic Mechanical Thermal Analysis (DMTA), Q800 from TA instruments. The glass transition temperature covers a wide temperature range from 60 to 120 °C.

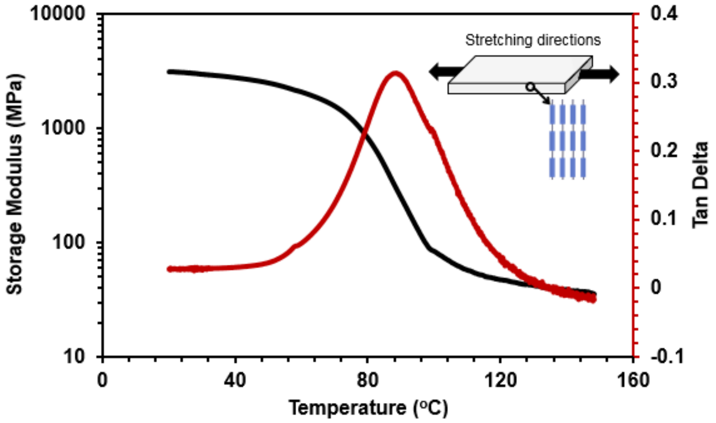

Supplementary Figure 5. Storage modulus and tan delta as a function of temperature of the polymer film. Stretching direction with respect to liquid crystal alignment in polymerized films (inset).

Supplementary Note 6: Molecular Simulation

Coarse-grained molecular dynamics simulations were carried out to examine the microscopic behavior of polymer networks with electrically-responsive mesogenic species. We focus on how the system volume and mesogen order parameter responds to an oscillating external electric field, relative to the system without the field.

The simulation system is a cubic box with volume V that can fluctuate, under constant pressure P. The system boundaries are periodic. Simulation parameters and quantities are all given in terms of fundamental reduced units of distance $\mathcal{D}$, energy $\mathcal{E}$, mass $\mathcal{M}$, and time $\tau =$

$\sqrt{\mathcal{M}\mathcal{D}^2/\mathcal{E}}$. Calculations were carried out using the HOOMD-Blue molecular dynamics package (v2.1.1).[3,4]

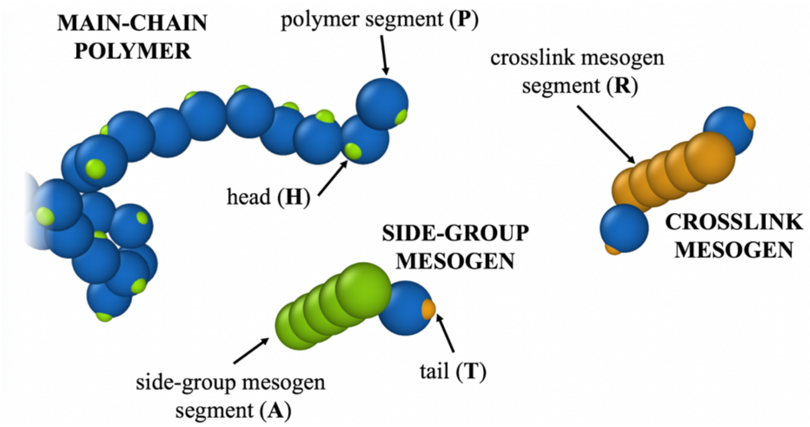

Supplementary Figure 6. Simulation representations of a polymer main chain, side-group mesogen, and crosslink mesogen. Polymer main chains are composed of **P** particles, each having one **H** particle, acting as a side-group or crosslink binding site. Crosslink mesogens are sequences of 5 **R** particles (collectively called a "mesogen"), terminated with two **P** particles each having a **T** particle that allows for binding to a main chain **H** particle. Side-group mesogens are sequences of 5 **A** particles, capped on one side by a **P** and **T** particle. All systems examined here contain 70 main chains each of 50 segments, 1166 crosslink mesogens, and the same number of side-chain mesogens.

The system consists of polymer main chains, connected together into a network with crosslinking mesogens. The main chains also have side-group mesogens. These are each illustrated in Supplementary Figure 6. The system contains no explicit or implicit solvent. All systems are integrated using a time step size of $dt = 0.001\tau$. Simulation approaches related to ours here are given in Refs. 5 and 6.

Simulations have been carried out for a variety of temperatures, electric field strengths, and field oscillation frequencies. These quantities are given in terms of simulation units as follows:

- Temperature T: $k_B T_{\mathrm{real}}/\mathcal{E}$
- Electric field strength E: $\sqrt{\mathcal{E}/(4\pi\epsilon_0\mathcal{D}^3)}$
- Electric field oscillation frequency f: $(100 \times dt)^{-1}$

- Simulation time $100 \times dt$

Here, ϵ_0 is the permittivity of vacuum, k_B is the Boltzmann constant, and T_{real} is real (experimental) temperature. The model ingredients and calculation methods are now described in the following sections.

Polymer main chains

Polymer main chains are represented as strings of particles **P**, held together by harmonic bonds. The polymer segments **P** have intermolecular pair-wise interactions obeying the Lennard-Jones potential:

$$U_{\text{LJ}}(r) = 4\epsilon\left[\left(\frac{\sigma}{r}\right)^{12} - \left(\frac{\sigma}{r}\right)^{6}\right] + C_{\text{shift}} \tag{1}$$

where r is the distance between the two particles. The potential is truncated at $r_{\text{cut}} = 2.5\mathcal{D}$; for larger r, the potential is zero. The constant C_{shift} is chosen such that $U_{\text{LJ}}(r_{\text{cut}}) = 0$. For **P**-**P** interactions, the segment size parameter $\sigma = 1\mathcal{D}$, and the interaction energy parameter $\epsilon = 1\mathcal{E}$. The harmonic bond potential connecting the polymer segments is

$$U_{\text{bond}}(r) = \frac{1}{2}k(r - r_0)^2 \tag{2}$$

The bond spring constant is set to $k = 1110\ \mathcal{E}/\mathcal{D}^2$ and the equilibrium bond distance to $r_0 = 0.9\ \mathcal{D}$.[7] The polymer has no angle or dihedral potentials.

Crosslink mesogens

A crosslink mesogen is represented by 5 Lennard-Jones particles **R**, held together by bonds of fixed length 0.5 $\mathcal{D}$. The two terminal **R** particles are each attached to a single **P** particle. The sequence of **R** particles is held into a rod-like configuration by 3-body angle potentials

$$U_{\text{ang}}(\theta) = \frac{1}{2} k_\theta (\theta - \pi)^2 \qquad (3)$$

where θ is the angle (in radians) between three adjacent **R** particles. (An angle of π corresponds to when the three particles are in a straight line). $U_{\text{ang}}(\theta)$ is applied to every three contiguous **R** particles in the sequence of 5 comprising the mesogen. Stiffness of the mesogen is enforced with $k_\theta = 200\ \mathcal{E}/\text{rad}^2$ in $U_{\text{ang}}(\theta)$. Intermolecular **R**-**P** and **R**-**R** interactions are given by $U_{\text{LJ}}(r)$ with $\sigma = 1\mathcal{D}$ and $\epsilon = 1\mathcal{E}$.

Side-chain mesogens

A side-chain mesogen is constructed from 5 Lennard-Jones "**A**" particles, connected by bonds of fixed length 0.5 $\mathcal{D}$. One of the two terminal **A** particles is bound to a single **P** particle. The same angle potentials $U_{\text{ang}}(\theta)$ as in the crosslink mesogens are applied here to enforce stiffness of the 5 **A** particles. Like the crosslink mesogens, intermolecular **A**-**A**, **A**-**R**, and **A**-**P** interactions are given by $U_{\text{LJ}}(r)$ with $\sigma = 1\mathcal{D}$ and $\epsilon = 1\mathcal{E}$.

Initial mesogen alignment & forming the polymer network

Polymer main chains, crosslink mesogens, and side-chain mesogens are placed into a simulation box with given initial fixed volume V_{xl}. To obtain a polymer network with built-in mesogen alignment, crosslinking of the system is performed in the presence of a "dummy" electric field along the *x* axis. Mesogens are driven to align parallel to the field vector by assigning equal but opposite electric charges q to their first and last **A** or **R** segments. The interaction of a charged **A** or **R** particle with the field, based on its coordinate *x* in the system, is given by

$$U_{\mathrm{EA}}(x) = -qEx \quad (4)$$

where E is the field magnitude, which can be varied to obtain different degrees of alignment in the final network. (Note that the system does not incorporate any Coulombic interactions between charged particles; the charge serves only to cause interaction with the imposed electric field.)

After equilibration in the NVE ensemble, the ingredients are crosslinked into a polymer network that percolates the system across its periodic boundaries in all three dimensions.

Crosslinking is carried out *in situ* as follows. Each polymer main chain segment is given a “head” particle **H**, and the terminal **P** segments on the side-chain and crosslink mesogens are each given a “tail” particle **T**. The **H** and **T** particles act as single binding sites on the polymer main chains and mesogens, respectively. The **H** and **T** particles are placed at fixed distances 0.4 $\mathcal{D}$ from the centers of their host **P** segments, and may freely rotate around the centers of their hosts. See Supplementary Figure 6 for a graphical depiction.

Intermolecular interactions are defined to enforce one-to-one **H-T** binding, i.e. such that each **H** can only be bound to one **T** at a given time, and vice-versa. First, a repulsive inverse power potential

$$U_{\mathrm{pow}}(r) = 4\epsilon\left(\frac{\sigma}{r}\right)^{12} + C_{\mathrm{shift}} \quad (5)$$

is defined between (**H**,**T**) particles, and (**P**, **R**, **A**) particles (with $\sigma = 0.6\ \mathcal{D}$, $\epsilon = 1\ \mathcal{E}$), as well as between **H**-**H** and **T**-**T** pairs ($\sigma = 0.9\ \mathcal{D}$, $\epsilon = 1\ \mathcal{E}$). The potential is cut off at $r_{\mathrm{cut}} = 2.5\mathcal{D}$, and C_{shift} is chosen to make $U_{\mathrm{pow}}(r_{\mathrm{cut}}) = 0$. Next, a strong attractive Gaussian potential between **H** and **T** particles is defined, given by

$$U_{\mathrm{gauss}}(r) = \epsilon_{\mathrm{HT}} \exp\left[-\frac{1}{2}\left(\frac{r}{\sigma_{\mathrm{HT}}}\right)^2\right] \quad (6)$$

in which $\epsilon_{\mathrm{HT}} = -500\ \mathcal{E}$ and $\sigma_{\mathrm{HT}} = 0.2\ \mathcal{D}$ are chosen. This potential is cut off at $r = 3\mathcal{D}$. Note that the parameters for $U_{\mathrm{pow}}(r)$ and $U_{\mathrm{gauss}}(r)$ are only relevant for the crosslinking process now described, as both potentials are removed from the simulation immediately afterwards.

To crosslink the system, we repeatedly perform short NVE evolutions of the system followed by crosslink formation. Crosslink formation consists of examining all **H** particles that are currently overlapping with **T** particles due to $U_{\mathrm{gauss}}(r)$. Between each overlapping **H-T** pair, a *permanent* bond of fixed length 0 is formed. The given **H** and **T** particles are then removed from the list of candidates for bond formation.

Cycles of NVE simulations followed by crosslinking are continued until a given percentage of **H** or **T** particles have been permanently bound to a partner. After this, the intermolecular interaction fields $U_{\mathrm{pow}}(r)$ and $U_{\mathrm{gauss}}(r)$ for **H** and **T** particles are removed.

Equilibration

After crosslinking, systems are equilibrated in the NPT ensemble (in the presence of the dummy electric field $U_{\mathrm{EA}}(x)$) with $P = 1\ \mathcal{E}/\mathcal{D}^3$ for 10^6 time steps, at temperature *T*. Integration is performed using the Martyna-Tobias-Klein barostat-thermostat[8] (*5*), with temperature and pressure coupling parameters of unity. This ensemble allows the system volume to fluctuate at constant pressure. All charges on the mesogens are removed, and a second NPT equilibration is performed for 2 x 10^6 time steps without the dummy electric field.

Oscillating electric field

Equal but opposite unity charges are assigned to the first and last **A** particle of each side-chain mesogen, representing their dipolarity in experiment. (The crosslink mesogens are

electrically inactive, and thus do not have charges added to them.) A time-varying electric field is imposed on the system along the y axis:

$$U_{\mathrm{EB}}(y,t) = -E'(t)y \tag{7}$$

The instantaneous field intensity $E'(t)$ is varied in time according to a desired profile. In this study, a sine wave oscillation of the field in experiment is approximated in simulation by a "sawtooth" profile. For a field with frequency f, the field intensity is varied in time according to

$$\frac{E\prime(t)}{E} = \begin{cases} 4tf,\ 0 \le t \le \frac{1}{4f} \\ -4\left(tf - \frac{1}{2}\right),\ \frac{1}{4f} < t \le \frac{3}{4f} \\ 4(tf-1),\ \frac{3}{4f} < t < \frac{1}{f} \end{cases} \tag{8}$$

where E is the max field intensity, and t is measured in units of $(100 \times dt)$. When t reaches $1/f$, t is reset to zero, so that the intensity profile is repeated periodically with a period of $1/f$. Note that the sign of the field intensity indicates whether the field direction is from $+y$ or $-y$.

Systems are integrated in the presence of the oscillating electric field in the NPT ensemble. Following an equilibration period of 2 x 10^4 time steps (within the field), results and statistics are obtained over 10^4 time steps.

Supplementary Movie 4 shows a system under an oscillating electric field ($T = 0.35$ units, $E = 30$ units, $1/f = 120$ units). The oscillating field is applied after the first 1/5 of the movie.

Volume response

The instantaneous volume of the system can be measured at any point during the simulation. We define the "% volume modulation" as

$$\% \text{ vol. mod.} = 100 \times \frac{V_f - \bar{V}_i}{\bar{V}_i} \tag{9}$$

where V_f is the instantaneous volume of the system in the presence of the oscillating electric field, and $\bar{V}_i$ is the average volume of the system without the field. $\bar{V}_i$ is obtained over 10^6 time steps before applying the electric field. The average percent volume modulation is obtained by calculating $\bar{V}_f$, the average system volume in the presence of the electric field. This is computed over 10^4 time steps (after equilibrating within the field for 2 x 10^4 time steps).

Mesogen order parameter

A vector $\mathbf{v_{rod}}$ is defined between the first and last particle (**A** or **R**) of each mesogen. The order parameter for one mesogen along the direction y of the electric field is computed by

$$\text{rod order parameter}(y) = \frac{1}{2}\left(3\cos^2\theta_{\mathbf{v}y} - 1\right) \tag{10}$$

where $\theta_{\mathbf{v}y}$ is the angle between $\mathbf{v_{rod}}$, and a unit vector along the y axis.

The average order parameter of the system is the mean of Supplementary Eq. 4 over all mesogens in the system. An average order parameter of -0.5 corresponds to when all mesogens are perpendicular to *y*; a value of 1 means that they are all aligned with *y*. Initial and final average mesogen order parameters in simulation were obtained over the same intervals of time steps as described in "Volume response" above.

System design & parameters

All systems in this work begin from the same large crosslinked network configuration. This network was created using 70 main chains each of 50 segments, 1166 crosslink mesogens, and the same number of side-chain mesogens. Thus, the system has one crosslink mesogen and one side-chain mesogen per every three main-chain segments on average.

The ingredients were placed into a simulation box with initial volume $V_{xl} = (35\,\mathcal{D})^3$, and crosslinked at $T = 0.35$. The crosslinking process was continued until at least 85% of the **H** or **T** particles had formed permanent bonds in the system. Crosslinking was also carried out with strong mesogen ordering along the x axis (i.e. perpendicular to the oscillating electric field axis y), resulting in an average order parameter of -0.42 along y at $T = 0.35$ after crosslinking was complete. A simulation snapshot of the system after equilibration is given in Supplementary Figure 7.

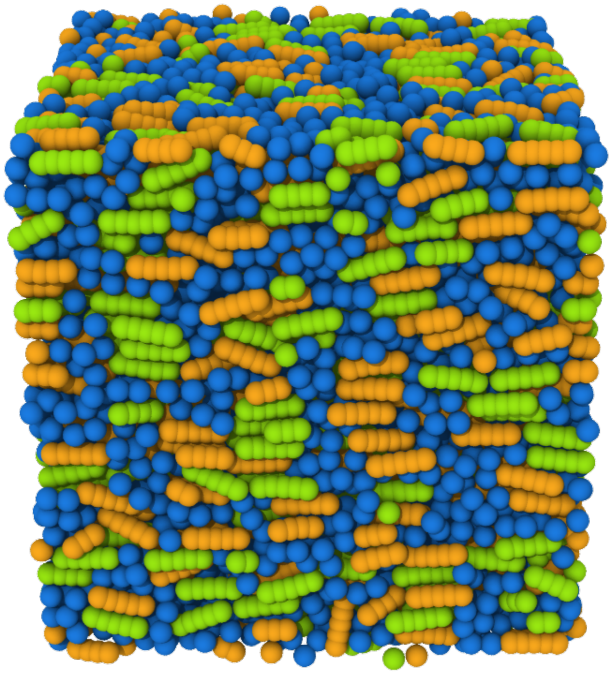

Supplementary Figure 7. Simulation snapshot of a system at $T = 0.35$ before the electric field is applied. Polymer main chain segments are shown in blue, crosslink mesogens in orange, and dipolar side-chain mesogens in green. For visual clarity, head (**H**) and tail (**T**) particles are not shown.

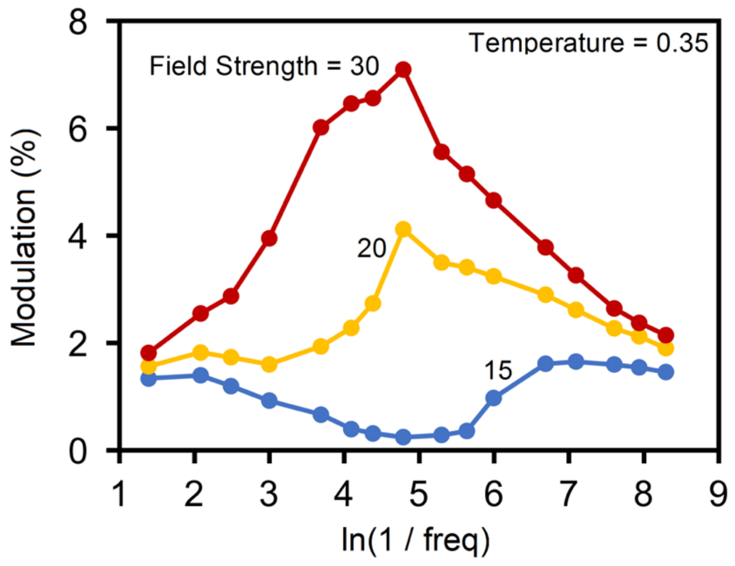

Supplementary Figure 8. Percent volume change of the simulated sample vs. electric field inverse frequency, for three choices of field strength at fixed $T = 0.35$ units. Points are simulation results, and lines are guides to the eye.

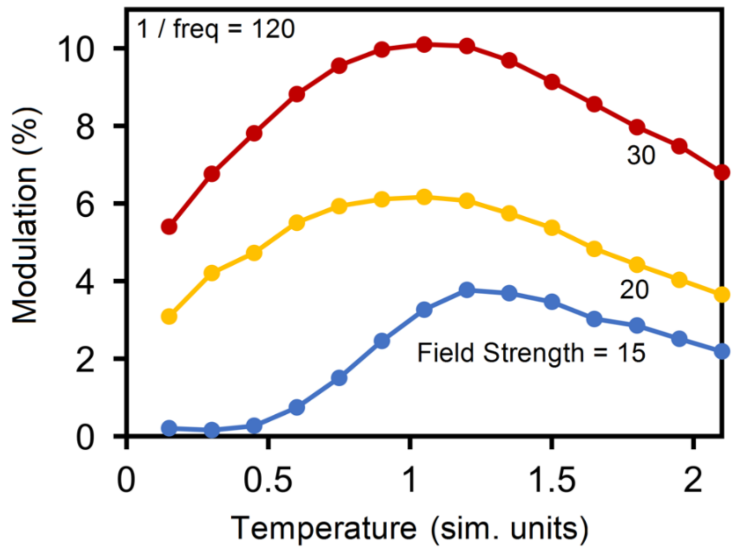

Supplementary Figure 9. Percent volume change of the simulated sample vs. temperature, for three choices of field strength at fixed $1/f = 120$ units. Points are simulation results, and lines are guides to the eye.

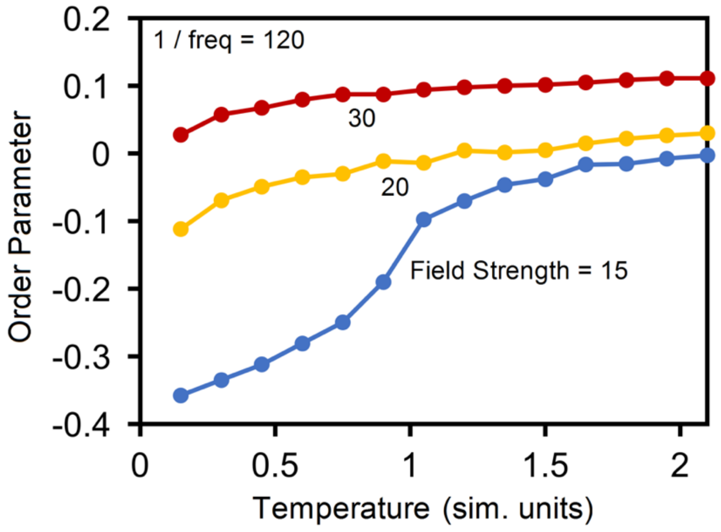

Supplementary Figure 10. Average mesogen order parameter (Supplementary Eq. 4) in the simulated sample vs. temperature, for three choices of field strength at fixed $1/f = 120$ units. Points are simulation results, and lines are guides to the eye. Recall that the initial equilibrated network at $T = 0.35$ has an average order parameter of -0.42.

Copies of this system were then equilibrated to different temperatures, and exposed to oscillating electric fields of different intensities and frequencies.

Supplementary results

Supplementary Figure 8 reports the percent volume modulation for systems all at fixed temperature $T = 0.35$, for different choices of field strength and oscillation frequency. At high field strength (30 units), the system exhibits a clear optimum oscillation frequency for the given sample temperature. Using an electric field at this frequency leads to large resonant volume fluctuations (orange curve, Figure 3C main text). At lower field intensity, this optimum frequency seems to correspond to a minimum in sample volume modulation.

The volume response of the network depends on the system temperature as well. In Supplementary Figure 9, we compare the volume modulation as a function of temperature for the same choices of field strength as in Supplementary Figure 8, all at the high-field-strength optimal

frequency for $T = 0.35$. At low temperatures, glassiness (i.e. low kinetic mobility) of the sample reduces the influence of the electric field in modulating the volume of the sample. This effect becomes particularly significant at low field intensities, relating to the shift in behavior of the volume response with field frequency in Supplementary Figure 8. At high temperatures, the polymer network is too mobile and fluid. Changes in local mesogen order therefore have a reduced impact on the overall sample volume.

Between the low- and high-temperature regimes is an optimum, at which the sample is sufficiently dense yet mobile to respond to changes in local mesogen order induced by the electric field. This optimum appears to be relatively constant with electric field strength, though the behavior of the system changes at low strengths.

Interestingly, the average order parameter of mesogens in the presence of the oscillating electric field *does not* correspond to the sample volume modulation. In Supplementary Figure 10, we find that the average order parameter in the system increases monotonically with temperature, without displaying an optimum like in Supplementary Figure 9. For the two higher field strength cases, the average order parameter varies little with system temperature. Instead, altering the field strength itself from 20 to 30 units has a more significant influence on the order parameter over the range of system temperatures shown here.

At low field strength, the behavior of the order parameter begins to deviate, displaying a more pronounced variation with temperature. Like in Supplementary Figures 8 and 9, a low electric field intensity in low-temperature samples only weakly modulates the mesogen alignment. In Supplementary Figure 10 this is evidenced by the more negative average order parameters relative to the higher-temperature systems (recalling that the average order parameter in the equilibrated network at $T = 0.35$ is -0.42 before the oscillating electric field is applied).

Supplementary References

1 Provided by Lyncée Tec SA (www.lynceetec.com).

2 Liu, D., Bastiaansen, C.W.M., Toonder, den, J.M.J. & Broer, D.J. Light-induced formation of dynamic and permanent surface topologies in chiral-nematic polymer networks. *Macromolecules* **45**, 8005-8012 (2012).

3 Anderson, J.A., Lorenz, C.D. & Travesset, A.. J. Comp. Phys. 227, 5342 (2008).

4 Glaser, J., Nguyen, T. D., Anderson, J. A. , Lui, P., Spiga, F., Millan, J. A., Morse, D. C. & Glotzer, S. C. Strong scaling of general-purpose molecular dynamics simulations on GPUs. *Comp. Phys. Comms.* **192**, 97 (2015).

5 Ilnytskyi, J. M., Saphiannikova, M., Neher, D. & Allen, M. P. Modelling elasticity and memory effects in liquid crystalline elastomers by molecular dynamics simulations. *Soft Matter* **8**, 11123-11134 (2012).

6 Darinskii, A.A., Zarembo, A., Balabaev, N.K., Neelov, I.M. & Sundholm, F. Molecular Dynamics Simulation of a Flexible Polymer Network in a Liquid Crystal Solvent: Dynamical Properties. *Polymer* **45**, 8901-8911(2004).

7 Peter, S., Meyer, H. & Baschnagel, J. Thickness-dependent reduction of the glass-transition temperature in thin polymer films with a free surface. *J. Poly. Sci. B: Poly. Phys.* **44**, 2951 (2006).

8 Martyna,G. J., Tobias, D.J. & Klein, M.L. Constant pressure molecular dynamics algorithms. *J. Chem. Phys.* **101**, 4177 (1994).
