# Supplementary material for: Protruding organic surfaces triggered by in-plane electric fields
Source: Nat Commun. 2017 Nov 15;8:1526. doi: 10.1038/s41467-017-01448-w (PMC5688145; doi:10.1038/s41467-017-01448-w)
Supplement: Supplementary file 3 — Description of Additional Supplementary Files [file 41467_2017_1448_MOESM3_ESM.pdf]

Description of Additional Supplementary Files

File Name: Supplementary Movie 1
Description: 3D view of formation of the surface topographies under the electric field. The AC field is switched on and off showing the changes in surface topography. Several cycles are shown. The movie is accelerated 4x.

File Name: Supplementary Movie 2
Description: Cross section profile of the formation of surface topographies. Cross-sectional deformation is derived from Supplementary Movie 1.

File Name: Supplementary Movie 3
Description: 3D view of formation of the surface topographies under the electric field. The coating follows the contour of the electrodes with deeper areas between the electrodes at zero voltage. Upon switching on the AC field the area between the electrodes grow higher. Several cycles are shown. The movie is accelerated 4x.

File Name: Supplementary Movie 4
Description: Oscillating effect of the protrusion. Deformation of one isolated protrusion derived from Supplementary Movie 2 showing a chaotic oscillating effect.

File Name: Supplementary Movie 5
Description: Simulated volume expansion under an AC field. Applying an oscillating electric field to the system causes the electrically-responsive mesogens to pivot, as they attempt to follow the shifting electric field vector. This movie shows the sample initially at rest, and then within the electric field. The oscillating field is applied after the first 1/5 of the movie. The total duration of the movie is 500 simulation time units. See the caption of Supplementary Figure 7 for colour details.
